# Upregulation of SERPINE2 Results in Poor Prognosis of Hepatoblastoma via Promoting Invasion Abilities

**DOI:** 10.1155/2022/2283541

**Published:** 2022-12-02

**Authors:** Guoyou Zou, Yong Lv, Meng Kong, Bo Xiang, Jing Chen

**Affiliations:** ^1^Department of Pediatric Surgery, West China Hospital, Sichuan University, Chengdu 610041, China; ^2^Department of General Surgery, People's Hospital of Tibet Autonomous Region, Tibet 850000, China; ^3^Laboratory of Pediatric Surgery, Frontiers Science Center for Disease-Related Molecular Network, West China Hospital, Sichuan University, Chengdu 610041, China

## Abstract

**Background:**

Hepatoblastoma (HB) is the most common malignant liver tumor in children. High-risk patients, especially those with tumor metastasis, have poor prognosis. Serpin family E member 2 (SERPINE2) is overexpressed in a variety of tumors, especially adenocarcinoma, and promotes tumor invasion and metastasis. The function and mechanism of SERPINE2 in HB are still unclear. The purpose of this study was to investigate the potential clinical prognostic value and molecular mechanism of SERPINE2 in HB.

**Methods:**

We performed bioinformatics analyses on HB microarray data GSE131329 to study the role of SERPINE2. The expression level of SERPINE2 in HB and its clinical significance were further analyzed by quantitative real-time polymerase chain reaction (qRT-PCR), Western blot, and immunohistochemistry. After constructing the SERPINE2 overexpression and knockdown in HepG2 and HUH6 cells, the 5-ethynyl-29-deoxyuridine (EdU) assay, wound healing assay, Transwell experiment, and apoptosis assay were performed to explore the role of SERPINE2 in HB progress.

**Results:**

Upregulation of SERPINE2 was found in HB tissues and was associated with a poor prognosis. Moreover, the SERPINE2 expression was related to tumor size, vascular invasion, and tumor metastasis. The Cox regressions show that high SERPINE2 expression is an independent risk factor for HB. SERPINE2 overexpression remarkably enhanced HB cell migration and invasion and suppressed apoptosis, while knockdown of SERPINE2 exerted the opposite effect. In addition, SERPINE2 facilitated the epithelial to mesenchymal transformation (EMT) phenotype of HB cells in vitro.

**Conclusion:**

Our findings indicated that SERPINE2 accelerates HB progression, suggesting that SERPINE2 may be a potential prognostic biomarker and an underlying therapeutic target for HB.

## 1. Introduction

Hepatoblastoma (HB) is the most common malignant tumor of liver in childhood [1]. In recent years, the incidence of HB is on the rise [2]. Most HB children have no obvious symptoms in the early stage and are not easy to detect. Most children with hepatoblastoma are diagnosed at a late stage when the disease has advanced to unresectable tumor. With the improvement of surgical techniques and adjuvant chemotherapy, the survival rate of HB children at low risk is close to 80%, but for high-risk patients, the overall survival rate is still very poor [3]. Currently, alpha-fetoprotein (AFP) is used as an indicator to detect the response to HB treatment and detect recurrent HB [4]. HB is associated with gene mutations such as catenin beta 1 (CTNNB1), NFE2-like BZIP transcription factor 2 (NFE2L2), MYC protooncogene (MYC), and yes-associated transcriptional regulator (YAP), and these gene tests may also be useful indicators for early diagnosis [5]. However, the specificity of these indicators is not enough. Improving the molecular mechanism of the disease and finding effective therapeutic targets are the most urgent needs for HB treatment.

Microarray technology is a widely used method to explore the pathogenesis of different diseases. This technology combined with protein interaction network can help to understand key genes and their interactions. The MCODE algorithm is very important to fully understand network properties and protein interaction network functions [6]. Through bioinformatics methods, SERPINE2 was identified as a candidate key gene. SERPINE2 is a glycoprotein and a member of the serpin family with serine protease inhibitory activity [7]. SERPINE2 has previously been reported to be involved in a variety of pathophysiological processes [8]. Recent studies have reported that SERPINE2 is overexpressed in a variety of tumors and promotes tumor progression, and SERPINE2 may be an oncogenic gene [9]. However, the mechanism of SERPINE2 in HB has not been reported.

In this study, we examined SERPINE2 expression in hepatoblastoma and its association with clinicopathological features. In addition, we also studied the molecular mechanism of hepatoblastoma development in HB cell lines. It provides a theoretical basis for finding new molecular biomarkers and effective therapeutic targets, which is of great clinical significance for improving the clinical prognosis of children with hepatoblastoma.

## 2. Material and Methods

### 2.1. Clinical Data

The study was approved by the Ethics Committee of West China Hospital of Sichuan University 2019(1085), and informed consent was obtained. Hepatoblastoma patients who underwent surgical treatment in the Department of Pediatric Surgery in West China Hospital of Sichuan University from January 2015 to December 2020 were collected. Inclusion criteria are as follows: (1) pathological diagnosis of hepatoblastoma; (2) age < 14 years old; and (3) preoperative radiotherapy, chemotherapy, or other adjuvant therapy were not received. Exclusion criteria are as follows: (1) complicated with severe organ dysfunction and (2) the patient requested to withdraw from the study or did not receive follow-up. A total of 66 children with complete clinical and pathological data were included in the study. Clinical data were collected, and Pretreatment Extent of Disease System (PRETEXT) stage was evaluated by abdominal ultrasound, abdominal CT, abdominal MRI, chest CT, etc. [10]. The COG stage was strictly in accordance with the standard of American Children's Oncology Group (COG) [11]. Through outpatient and telephone follow-up, the patients were followed up to December 2021, with a median follow-up time of 33 months, and 4 cases were lost to follow-up.

### 2.2. Microarray Data Information and Hub Gene Screen

We use the keywords “hepatoblastoma” from Gene Expression Omnibus (GEO) repository (http://www.ncbi.nlm.nih.gov/geo) and download the data associated with HB. We obtained the gene expression profile of GSE131329 from GEO database; GSE131329 included 39 primary hepatoblastoma tissues, 14 metastasis hepatoblastoma tissues, and 14 normal liver tissues. The differentially expressed genes (DEGs) were filtered via the R software with *P* < 0.05 [12]. WGCNA is a systematic biological method for characterizing gene association patterns between different samples and can be used to identify highly synergistic sets of genes and to screen for candidate biomarker genes or therapeutic targets [13]. We used the WGCNA function package in R software to screen for gene modules associated with disease phenotypes and further analyzed the relationship between these modules and HB tumor samples. We quantify associations of individual genes with our trait of interest (tumor metastasis) by defining gene significance (GS) as the correlation between the gene and the trait. For each module, we also define a quantitative measure of module membership (MM) as the correlation of the module eigengene and the gene expression profile. This allows us to quantify the similarity of all genes on the array to every module [14]. The protein-protein interaction (PPI) network was obtained by STRING (https://cn.string-db.org/), and the MCODE plug-in was used to screen the hub gene of the PPI network with the following parameter settings: node score cutoff = 0.2, degree cutoff = 2, maximum depth = 100, and k − core = 2 [15].

### 2.3. Quantitative Real-Time Polymerase Chain Reaction (qRT-PCR)

TRIzol method was used to extract total RNA from HB tumor and adjacent tissues, and cDNA was obtained after removing genomic DNA. PCR amplification was performed using SYBR reagent (Genecopeia, USA) in the Bio-Rad CFX Connect fluorescence quantitative PCR instrument. The relative expression levels were calculated by 2−*ΔΔ*Ct method. Primers were designed according to the CDS sequence of SERPINE2:

1. SERPINE2-F: 5′-AAGAAACGCACTTTCGTGGC-3′
2. SERPINE2-R: 5′-GTGTGGGATGATGGCAGACA-3′
3. GAPDH-F: 5′-GGTGGTCTCCTCTGACTTCAACA-3′
4. GAPDH-R: 5′-TTTGCTGTAGCCAAATTCGTTGT-3′

### 2.4. Immunohistochemistry (IHC)

Paraffin-embedded tissue slides were heated, dewaxed, and dehydrated, then repaired with H_2_O_2_ and citric acid antigen, and sealed with goat serum, and polyclonal SERPINE2 rabbit primary antibody (1 : 50; GeneTex, USA) was incubated overnight (PBS was used instead of primary antibody as negative control); goat anti-rabbit secondary antibody was incubated the next day and incubated at room temperature for 60 min. The tissue sections were rinsed with PBS for 3 times; DAB color solution was added and observed under an optical microscope. (1) Staining intensity scoring criteria are as follows: 0 for no staining, 1 for yellow, 2 for brownish yellow, and 3 for tawny brown. (2) The scoring criteria for the proportion of positive cells are as follows: 0 points for the number of positive cells < 5%, 1 point for the number of positive cells between 6% and 25%, 2 points for the number of positive cells between 26% and 50%, 3 points for the number of positive cells between 51% and 75%, and 4 points for the number of positive cells over 75%. When we multiply the above two scores, we get 0-1 (-), 2-4 (+), 5-8 (++), and 9-12 (+++). Low expression was defined as a final score of <5 and the rest as high expression.

### 2.5. Cell Culture

HB cell lines HepG2 and Huh6 (both purchased from Cell Bank of Chinese Academy of Sciences (CSTR: 19375.09.3101HUMSCSP510 and CSTR: 19375.09.3101HUMTCHu181) were cultured in DMEM basal medium containing 10% fetal bovine serum, 100 U/mL penicillin, and 100 *μ*g/mL streptomycin. The cells were placed in a cell incubator containing 5% CO_2_ and 95% humidification at 37°C. After the cell fusion rate reached 90%, the cells were digested and passed by 0.25% trypsin for subsequent experiments.

### 2.6. RNA Interference and Overexpression of SERPINE2

Small interfering RNA (siRNA) to suppress the expression of SERPINE2 was purchased from Beijing Optimus Biotechnology Co., Ltd. (Beijing, China). The CDS region of SERPINE2 was amplified by PCR from the cDNA of HB tumor tissue and linked to the PCS2-C-myc vector to construct the overexpressed plasmid (OE-SERPINE2). The empty vector (PCS2-C-myc) was used as a control (OE-NC). According to the manufacturer's instructions, siRNA and plasmid were transfected into HepG2 and Huh6 cell lines using lipo8000TM (Beyotime C0533) transfection reagent (Beyotime Shanghai, China), and SERPINE2 was knocked down and overexpressed.

### 2.7. 5-Ethynyl-29-Deoxyuridine (EdU) Assay

Cell proliferation was measured by EdU method after transfection. The assays were performed as recommended by the manufacturer of EdU detection kits. Then, BD-FACSCanto II flow cytometry was used to analyze the proliferation rate of each treatment group.

### 2.8. Apoptosis Assay

For detection of cell apoptosis, the transfected cells were harvested, the Annexin V-FITC and PI were successively added for staining in the dark room according to the instructions of Annexin V/PI kit. The cells were analyzed by flow cytometry using BD-FACSCanto II flow cytometry. FlowJo-V10 software is used for data collection and processing.

### 2.9. Scratch Assay

The migration ability of HB cells was detected by scratch assay. The transfected cells were scratched with 20 *μ*L sterile spear tip, washed twice with PBS, and cultured in new DMEM medium (excluding FBS). Photos were taken at the same observation point at 0 h, 24 h, and 48 h under the microscope.

### 2.10. Transwell Assay

Transfected cells were collected, the cell concentration was adjusted to 5 × 10^5^/mL, and 100 *μ*L cell suspension was inoculated into the upper chamber of labselt-14341 (Beijing, China), and 700 *μ*L DMEM medium (containing 10% FBS) was added to the lower chamber. After 24 hours of coculture, the chamber was carefully removed and stained with fast Reischel-Giemsa staining kit. Nine fields were randomly selected to count. In the invasion experiment, a layer of Matrigel (Corning CAT: 356243) was laid on the bottom membrane of the upper compartment before the cells were inoculated, and the other procedures were the same as the migration experiment.

### 2.11. Western Blot (WB)

Transfected cells were collected, total protein was extracted by TNE lysis, and protein concentration was determined using BCA protein concentration determination kit (Beyotime, Shanghai, China). The extracted protein was separated with SDS-PAGE, and the isolated protein was transferred to PVDF membranes (Millipore, Billerica, MA, USA). The membranes were blocked in 5% skim milk, primary antibody (SERPINE2 antibody, Art. GXT124069, 1 : 1500; E-cadherin antibody, GXT100443, 1 : 2000; N-cadherin antibody, GXT127345, 1 : 1500; and internal reference *β*-actin, A1978, 1 : 3,000) was incubated overnight, the membrane was washed for three times for secondary antibody (goat versus rabbit), and the membranes were analyzed under a Bio-Rad image analysis system.

### 2.12. Statistical Analysis

The SPSS 22.0 software (IBM USA) was used for data analysis. The chi-square test was used for categorical variables, and Student's t-test was used for continuous variables. The Kaplan-Meier method was used to draw survival curves, and the log-rank test was used to compare the survival differences of different variables. Independent prognostic indicators of overall survival were used in a multivariable Cox regression analysis. A double-sided *P* value < 0.05 was considered statistically significant.

## 3. Result

### 3.1. Bioinformatics Analysis and Verification of SERPINE2 in Hepatoblastoma

**Figure 1:**
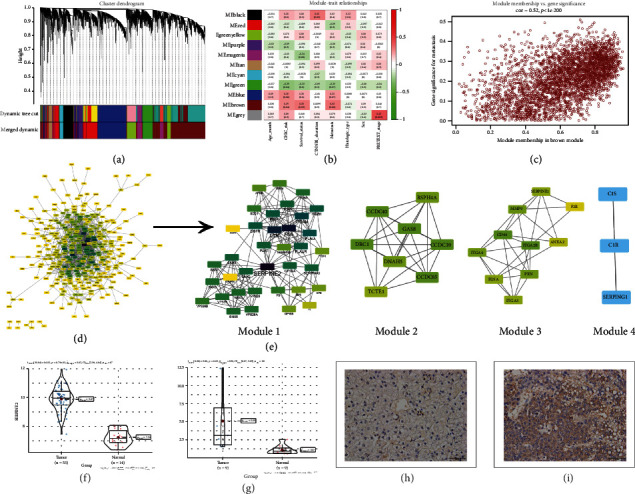
Bioinformatics analysis and SERPINE2 expression in hepatoblastoma. (a) The cluster dendrogram of genes. Genes that could not be clustered into one of these modules were assigned to the gray module. Every gene represents a line in the hierarchical cluster. (b) Heatmap of the module-trait relationships. (c) Scatterplots of gene significance (GS) versus module significance (MS) in brown modules. (d) PPI networks show the interaction of genes related to HB metastasis. The nodes and edges are retrieved from the STRING tool and plotted using Cytoscape software. (e) The four cluster subnetworks were identified from the PPI network with the help of Cytoscape using the MCODE plug-in with a cluster score. (f) The expression of SERPINE2 in hepatoblastoma and normal liver in GSE131329. (g) The expression of SERPINE2 in hepatoblastoma and matched nontumor liver tissues in our hospital. (h) Immunohistochemistry detection of SERPINE2 expression in hepatoblastoma adjacent normal liver tissues. (Representative images were shown at ×40 (scale bar, 50 mm) via microscope.) (i) Immunohistochemistry detection of SERPINE2 expression in hepatoblastoma tissues. (Representative images were shown at ×40 (scale bar, 50 mm) via microscope.)

According to the cutoff criteria for selecting DEGs, a total of 7,468 DEGs were recognized between metastatic hepatoblastoma and normal liver. The expression profiles of these DEGs were analyzed using the WGCNA package in R software. We obtained 11 different gene modules from WGCNA (Figure 1(a)). The brown module was the gene module most associated with HB metastasis (Figure 1(b)). The brown gene module contains 3,639 genes. Because tumor metastasis was associated with poor prognosis in HB patients, we selected the genes in the brown module for subsequent analysis. A total of 423 genes with high similarity and significance (module membership > 0.8 and gene significance > 0.2) were screened in the brown module as candidate hub genes (Figure 1(c)). The 423 genes were associated with HB metastasis. And then, the 423 genes were input into the PPI network to explore the modules available for exploring HB metastasis-related genes; the results showed that there were 331 nodes and 1,077 edges in the PPI network (Figure 1(d)). Based on the PPI network and MCODE plug-in, there were four modules in the PPI network, and the scores of the four modules were as follows: 10.343 (module 1), 7.429 (module 2), 6.000 (module 3), and 3.000 (module 4), respectively. The first module (module 1) with the highest scores is located at the center of the whole network, including 36 nodes and 181 edges (Figure 1(e)). Therefore, SERPINE2, with the highest degree, which rank the first in module 1, maybe the most important part of the whole network. To confirm whether SERPINE2 is associated with the development of hepatoblastoma, we examined the expression level of SERPINE2 in hepatoblastoma tissues. The SERPINE2 mRNA expression levels were higher in hepatoblastoma tissues than in normal liver tissues in the GSE131329 dataset (Figure 1(c)). In addition, we obtained the SERPINE2 mRNA expression from 9 pairs of hepatoblastoma tissues and matched nontumor liver tissues in our laboratory. The qRT-PCR results showed that SERPINE2 mRNA expression level in tumor tissues was significantly higher than that in the corresponding liver tissues (Figure 1(d)). We used immunohistochemistry to detect SERPINE2 expression in 66 pairs of hepatoblastoma tumor tissues and matched nontumor liver tissues (Figures 1(e) and 1(f)). The SERPINE2-positive rate was 86.4% (57/66) in HB tissue and 18.2% (12/66) in adjacent liver tissue; the difference was statistically significant (*P* < 0.05).

### 3.2. SERPINE2 Is Correlated with Malignancies in Hepatoblastoma

We used hepatoblastoma tissue in our hospital to explore the clinical value of SERPINE2. Detailed clinical characteristics of 66 HB patients are summarized in Table 1. We used IHC staining to explore the associations between SERPINE2 expression and the clinicopathological features of HB patients. The SERPINE2 expression score < 5 was defined as low expression group, and ≥5 was defined as high expression group; 66 HB patients were divided into high expression group (*n* = 43) and low expression group (*n* = 23). As shown in Table 1, the high expression of SERPINE2 was closely related to tumor size (*P* = 0.021), vascular invasion (*P* = 0.029), tumor metastasis (*P* = 0.039), and PRETEXT stage (*P* = 0.049). These results suggest that SERPINE2 may play an important role in the invasion and metastasis of hepatoblastoma.

### 3.3. High Expression of SERPINE2 Is Associated with Poor Outcomes in HB Patients

**Figure 2:**
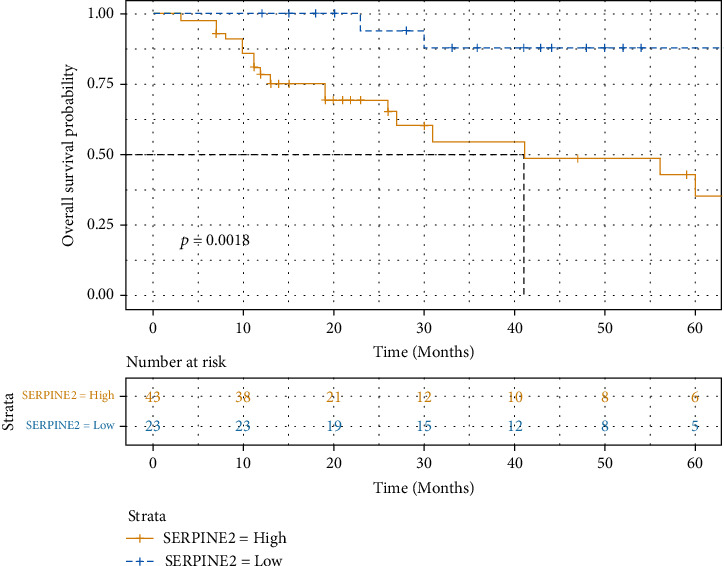
SERPINE2 expression the hepatoblastoma cohort using the cutoff (low (IHC score < 5) vs. high (IHC score ≥ 5)) as a determinant of overall survival in the Kaplan-Meier analysis (*P* < 0.001, log-rank test). IHC: immunohistochemistry.

In view of the correlation between the expression level of SERPINE2 and tumor invasion and metastasis, we speculate that the expression level of SERPINE2 is also correlated with the survival and prognosis of patients. Therefore, we combined the follow-up data of HB patients to evaluate the relationship between SERPINE2 expression and overall survival of HB patients. The Kaplan-Meier survival curve showed that the overall survival time was shorter in the SERPINE2 high expression group (Figure 2). The mean postoperative survival time of the SERPINE2 high expression group was 48.34 months (95% CI: 35.94-60.73), and the 3-year survival rate was 60.47%. The mean postoperative survival time of the low expression group was 71.04 months (95% CI: 63.27-78.81), and the 3-year survival rate was 91.30%. The survival rate of SERPINE2 high expression group was significantly lower than that of SERPINE2 low expression group; the difference was statistically significant (*P* < 0.001). Univariate Cox analysis showed that vascular invasion (HR: 3.43, *P* = 0.012), tumor metastasis (HR: 6.72, *P* < 0.001), PRETEXT stage (HR: 7.46, *P* < 0.001), and SERPINE2 (HR: 7.36, *P* < 0.008) were the prognostic risk factors for hepatoblastoma. Multivariate Cox analysis showed that tumor metastasis (HR: 3.84, *P* < 0.006), PRETEXT stage (HR: 4.57, *P* < 0.011), and SERPINE2 (HR: 4.71, *P* < 0.040) were the independent prognostic risk factors for hepatoblastoma (Table 2).

### 3.4. SERPINE2 Significantly Inhibits HB Cell Apoptosis

**Figure 3:**
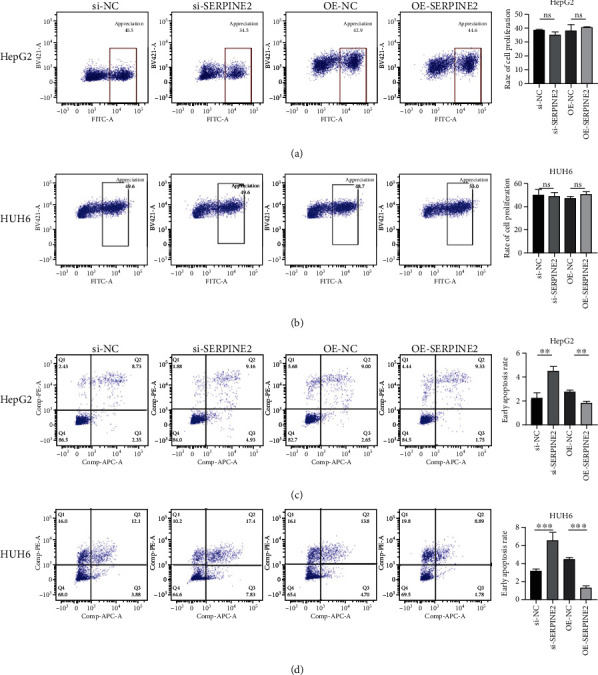
SERPINE2 did not significantly affect HB proliferation but significantly inhibited early apoptosis in HepG2 and HUH6 cells. (a) EdU assays detect the proliferation ability of HepG2 cells in OE-SERPINE2 group and si-SERPINE2 group. (b) EdU assays detect the proliferation ability of HUH6 cells in OE-SERPINE2 group and si-SERPINE2 group. (c) Flow cytometry detected the apoptosis rate of HepG2 cells in OE-SERPINE2 group and si-SERPINE2 group. (d) Flow cytometry detected the apoptosis rate of HUH6 cells in OE-SERPINE2 group and si-SERPINE2 group.

To explore the function of SERPINE2 in HB progression, we transfected OE-SERPINE2 plasmid and si-SERPINE2 into HepG2 and HUH6 cells and measured SERPINE2 mRNA and protein levels 48 hours later. The overexpression and knockdown efficiencies were verified by qRT-PCR and Western blot analysis (supplementary figure 1). The EdU assay showed that SERPINE2 did not significantly affect HB cell proliferation in vitro (Figures 3(a) and 3(b)). Apoptosis experiments showed that overexpression of SERPINE2 inhibited early apoptosis in HepG2 and HUH6 cells, and knockdown of SERPINE2 promoted early apoptosis (Figures 3(c) and 3(d)).

### 3.5. SERPINE2 Promotes the Migration and Invasion of HB Cells

**Figure 4:**
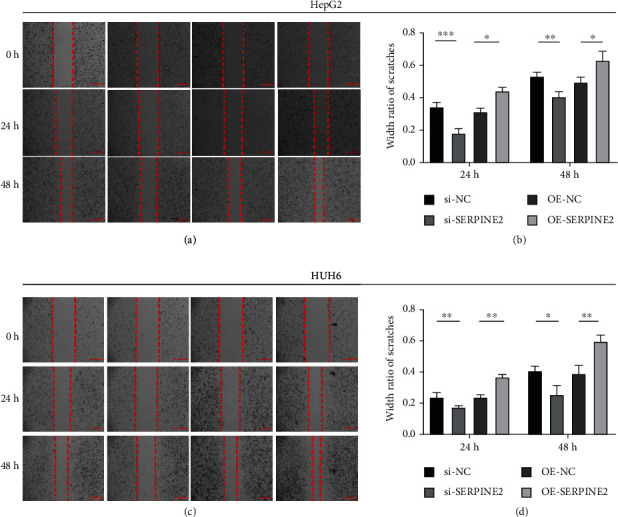
Wound healing assay. (a, b) Wound healing assay detects the migration ability of HepG2 cells in OE-SERPINE2 group and si-SERPINE2 group. (c, d) Wound healing assay detects the migration ability of HUH6 cells in OE-SERPINE2 group and si-SERPINE2 group.

**Figure 5:**
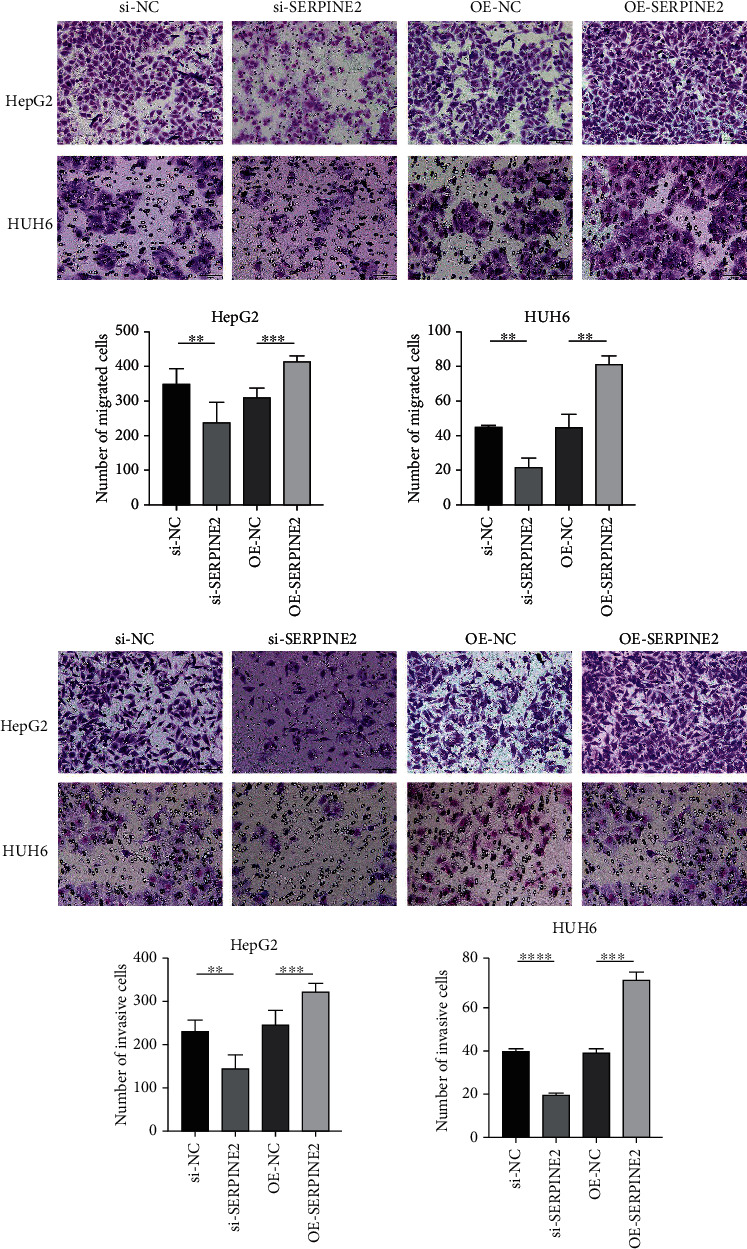
Transwell assay detects the migration and invasion ability of HB cells.

Scratch assay showed that in HepG2 and HUH6 cell lines, the wound healing rate of transfected si-SERPINE2 group was significantly lower than that of the control group, and the wound healing rate of OE-SERPINE2 group was significantly higher than that of the control group (Figure 4). Transwell migration experiment showed that the number of migrated cells in the OE-SERPINE2 group was significantly lower than that in the control group, and the number of migrated cells in the OE-SERPINE2 group was significantly higher than that in the control group (Figure 5(a)). Transwell invasion assay showed that SERPINE2 overexpression markedly enhanced invasive efficiencies of HB cells in vitro compared with the control empty vector transfected cells, whereas the knockdown of endogenous SERPINE2 significantly inhibited invasion (Figure 5(b)).

### 3.6. SERPINE2 Promotes Epithelial to Mesenchymal Transformation of HB Cells

**Figure 6:**
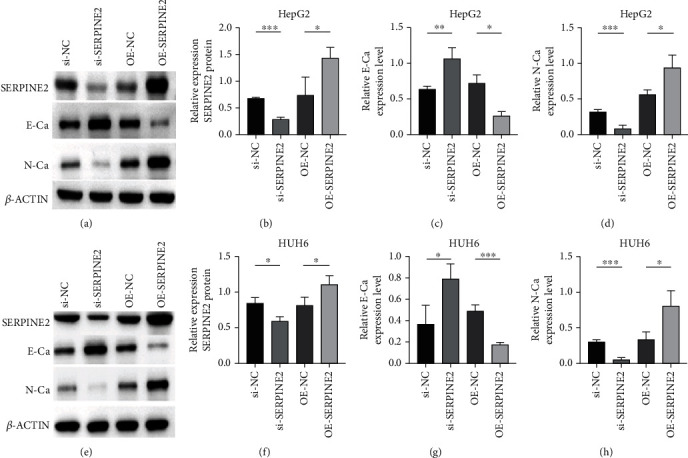
The effects of SERPINE2 on epithelial-mesenchymal transition in HB cells. (a–d) Western blot analysis of E-cadherin and N-cadherin in HepG2 cells transfected with OE-SERPINE2 group and si-SERPINE2 group. (e–h) Western blot analysis of E-cadherin and N-cadherin in HUH6 cells transfected with OE-SERPINE2 group and si-SERPINE2 group.

In view of the correlation between the expression level of SERPINE2 and tumor invasion and metastasis, we speculated that SERPINE2 might be involved in epithelial-mesenchymal transition. Therefore, we used Western blot to detect epithelial-mesenchymal transition-related indicators. Knockdown SERPINE2 induced early apoptosis of HB cell lines, SERPINE2 protein expression was decreased, E-Ca increased, and N-Ca decreased. SERPINE2 overexpression increased SERPINE2 protein expression, decreased E-Ca, and increased N-Ca (Figure 6), suggesting that SERPINE2 may promote epithelial to mesenchymal transformation.

## 4. Discussion

Hepatoblastoma is a common liver tumor in children, and the pathogenesis is unknown; transcriptome analysis and bioinformatics may give us an insight into the overall mechanism of HB [16]. Since bioinformatics can give gene expression levels in the human genome simultaneously, it has been frequently utilized to find diagnostic or prognostic biomarkers [17]. In this study, we used the weighted gene coexpression network analysis for the first time to screen out genes related to hepatoblastoma metastasis, and then, these genes were uploaded to STRING database. The protein interaction network among these genes was created using Cytoscape tools and MCODE app. After computation, SERPINE2 is considered as hub gene. The adhesion of tumor cells to extracellular matrix components can activate or secrete proteolytic enzymes to promote matrix degradation, thus forming a local lysis zone, which constitutes a pathway for tumor cell metastasis. SERPINE2 affects tumor invasion and migration through regulation of matrix metalloproteinases and plasminase systems [18]. Our study found that the expression level of SERPINE2 in HB tumor tissues was significantly higher than that in adjacent tissues, suggesting that SERPINE2 may be the oncogenic gene of HB. We found that high SERPINE2 expression was associated with poor prognosis of HB by IHC analysis. The high expression of SERPINE2 was related to tumor size, vascular invasion, tumor metastasis, and PRETEXT stage. Moreover, our data indicated that elevated expression of SERPINE2 acts as an independent prognostic biomarker of poor overall survival (OS) in patients with hepatoblastoma. These results are similar to the previous studies. SERPINE2 is differentially expressed in many tumors and their corresponding normal tissues and is highly expressed in adenocarcinoma, especially enriched in glandular organs of the digestive system and highly expressed in liver tumors [19]. Therefore, SERPINE2 may be a potential prognostic indicator of HB.

Apoptosis is the spontaneous and ordered death of cells controlled by genes. Previous studies have shown that SERPINE2 is closely associated with tumor cell apoptosis and malignant transformation [20]. Silencing SERPINE2 can induce apoptosis in endometrial cancer cells [21]. Knockdown of SERPINE2 in human papillary thyroid cancer cells decreased the antiapoptotic protein Bcl-2 and increased the proapoptotic protein Bax and caspase-3, thereby promoting cell apoptosis [22]. Our results also support this conclusion; knockdown SERPINE2 induces early apoptosis of HB cells, while overexpression of SERPINE2 enhances HB cell viability and inhibits early apoptosis. SERPINE2 may be an important regulator of HB apoptosis.

Metastasis is an important cause of poor prognosis of HB, and a large number of previous studies have reported that SERPINE2 is closely related to tumor invasion and metastasis. SERPINE2 is closely associated with the depth of invasion and lymph node metastasis of esophageal squamous cell carcinoma and promotes the migration and invasion of esophageal carcinoma cells by inducing EMT [23]. SERPINE2 is overexpressed in gastric cancer and is associated with poor survival, promoting gastric cancer cell migration and invasion [24]. SERPINE2 promotes melanoma metastasis through the glycogen synthesis kinase 3*β* (GSK-3*β*) signaling pathway [25]. In pancreatic cancer, SERPINE2 promotes pancreatic cancer invasion by promoting extracellular matrix deposition [26]. Our study also showed that SERPINE2 expression in HB was related to vascular infiltration and metastasis. SERPINE2 may promote the migration and invasion of HB cells by promoting EMT in vitro. SERPINE2 plays an important role in regulating extracellular matrix metabolism and is involved in the invasion, migration, and apoptosis of hepatoblastoma.

In conclusion, we found that SERPINE2 was correlated with poor prognosis in hepatoblastoma. SERPINE2 promotes HB tumor progression by inhibiting apoptosis and promoting migration and invasion of HB cells. We found that SERPINE2 plays an important role in HB progression, might represent a novel therapeutic strategy, and might be used as a prognostic marker of HB.

## Figures and Tables

**Table 1:** Relationship between SERPINE2 expression and hepatoblastoma clinicopathological features.

Characteristics	SERPINE2 expression		Overall	*P* value
	Low	High		
	(*N* = 23)	(*N* = 43)	(*N* = 66)	
Gender				
Male	15 (65.2%)	21 (48.8%)	36 (54.5%)	0.203
Age (months)				
Mean (SD)	57.4 (28.3)	50.1 (19.0)	52.7 (22.7)	0.214
Tumor size				
≥10 cm	6 (26.1%)	24 (55.8%)	30 (45.5%)	0.021
Histology type				
Mixed	9 (39.1%)	10 (23.3%)	19 (28.8%)	0.175
Vascular invasion				
Present	4 (17.4%)	19 (44.2%)	23 (34.8%)	0.029
Portal vein thrombus				
Present	2 (8.7%)	3 (7.0%)	5 (7.6%)	0.801
Tumor metastasis				
Present	3 (13.0%)	16 (37.2%)	19 (28.8%)	0.039
PRETEXT stage				
III-IV	7 (30.4%)	24 (55.8%)	31 (47.0%)	0.049
COG stage				
III-IV	7 (30.4%)	18 (41.9%)	25 (37.9%)	0.362
AFP				
<100 ng/mL	1 (4.3%)	2 (4.7%)	3 (4.5%)	0.955
Albumin(g/L)				
Mean (SD)	40.4 (5.3)	42.0 (5.0)	41.5 (5.2)	0.239
Total bilirubin (umol/L)				
Mean (SD)	12.4 (8.1)	14.7 (11.6)	13.9 (10.5)	0.402
Direct bilirubin (umol/L)				
Mean (SD)	5.4 (4.2)	7.9 (13.7)	7.1 (11.3)	0.404
Creatinine (umol/L)				
Mean (SD)	53.2 (27.1)	43.1 (24.6)	46.6 (25.8)	0.13
INR				
Mean (SD)	0.87 (0.20)	0.91 (0.17)	0.89 (0.18)	0.371
Platelet (10^9^/L)				
Mean (SD)	252 (132)	243 (131)	246 (131)	0.779
Prothrombin time(s)				
Mean (SD)	11.1 (1.2)	11.0 (1.9)	11.0 (1.7)	0.864

**Table 2:** Univariate and multivariate Cox proportional hazards analyses for the overall survival of hepatoblastoma.

Characteristic	Univariate			Multivariate		
	HR	95% CI	*P* value	HR	95% CI	*P* value
Gender						
Male vs. female	0.52	(0.22-1.16)	0.148			
Tumor size						
≥10 cm vs. <10 cm	1.48	(0.63-3.50)	0.368			
Histology type						
Mixed vs. epithelial	1.31	(0.52-3.30)	0.568			
Vascular invasion						
Present vs. absent	3.43	(1.31-8.98)	0.012			
Portal vein thrombus						
Present vs. absent	2.46	(0.54-11.20)	0.243			
Metastasis						
Present vs. absent	6.72	(2.66-16.96)	<0.001	3.84	(1.47-9.99)	0.006
PRETEXT stage						
III-IV vs. I-II	7.46	(2.44-22.80)	<0.001	4.57	(1.41-14.76)	0.011
COG stage						
III-IV vs. I-II	1.95	(0.82-4.65)	0.13			
AFP						
<100 (ng/mL) vs. ≥100 (ng/mL)	2.66	(0.33-21.4)	0.357			
SERPINE2						
High vs. low	7.36	(1.70-31.96)	0.008	4.71	(1.07-20.71)	0.04

## Data Availability

The datasets and materials used in this study are available on reasonable request.

## Abbreviations

HB: Hepatoblastoma
SERPINE2: Serpin family E member 2
EMT: Epithelial–mesenchymal transition
AFP: Alpha-fetoprotein
CTNNB1: Catenin beta 1
NFE2L2: NFE2-like BZIP transcription factor 2
MYC: MYC protooncogene
YAP: Yes-associated transcriptional regulator
PRETEXT: Pretreatment Extent of Disease System
COG: American Children's Oncology Group
GEO: Gene Expression Omnibus
DEGs: Differentially expressed genes
PPI: Protein-protein interaction
qRT-PCR: Quantitative real-time polymerase chain reaction
IHC: Immunohistochemistry
siRNA: Small interfering RNA
EdU: 5-Ethynyl-29-deoxyuridine
WB: Western blot
OS: Overall survival.

## Supplementary Materials

Supplementary Materials Supplementary figure 1: the relative mRNA and protein expression in HepG2 and HUH6 cell lines after being transfected with small interfering RNAs (siRNAs) and overexpressed plasmid against SERPINE2. (A) The relative mRNA expression in HepG2 cell lines after being transfected with small interfering RNAs (siRNAs) and overexpressed plasmid against SERPINE2. (B) The relative mRNA expression in HUH6 cell lines after being transfected with small interfering RNAs (siRNAs) and overexpressed plasmid against SERPINE2. (C, E) The relative protein expression in HepG2 cell lines after being transfected with small interfering RNAs (siRNAs) and overexpressed plasmid against SERPINE2. (D, F) The relative protein expression in HUH6 cell lines after being transfected with small interfering RNAs (siRNAs) and overexpressed plasmid against SERPINE2.
