# Supplementary figures and images for: Upregulation of SERPINE2 Results in Poor Prognosis of Hepatoblastoma via Promoting Invasion Abilities
Source: Dis Markers. 2022 Dec 2;2022:2283541. doi: 10.1155/2022/2283541 (PMC9734000; doi:10.1155/2022/2283541)

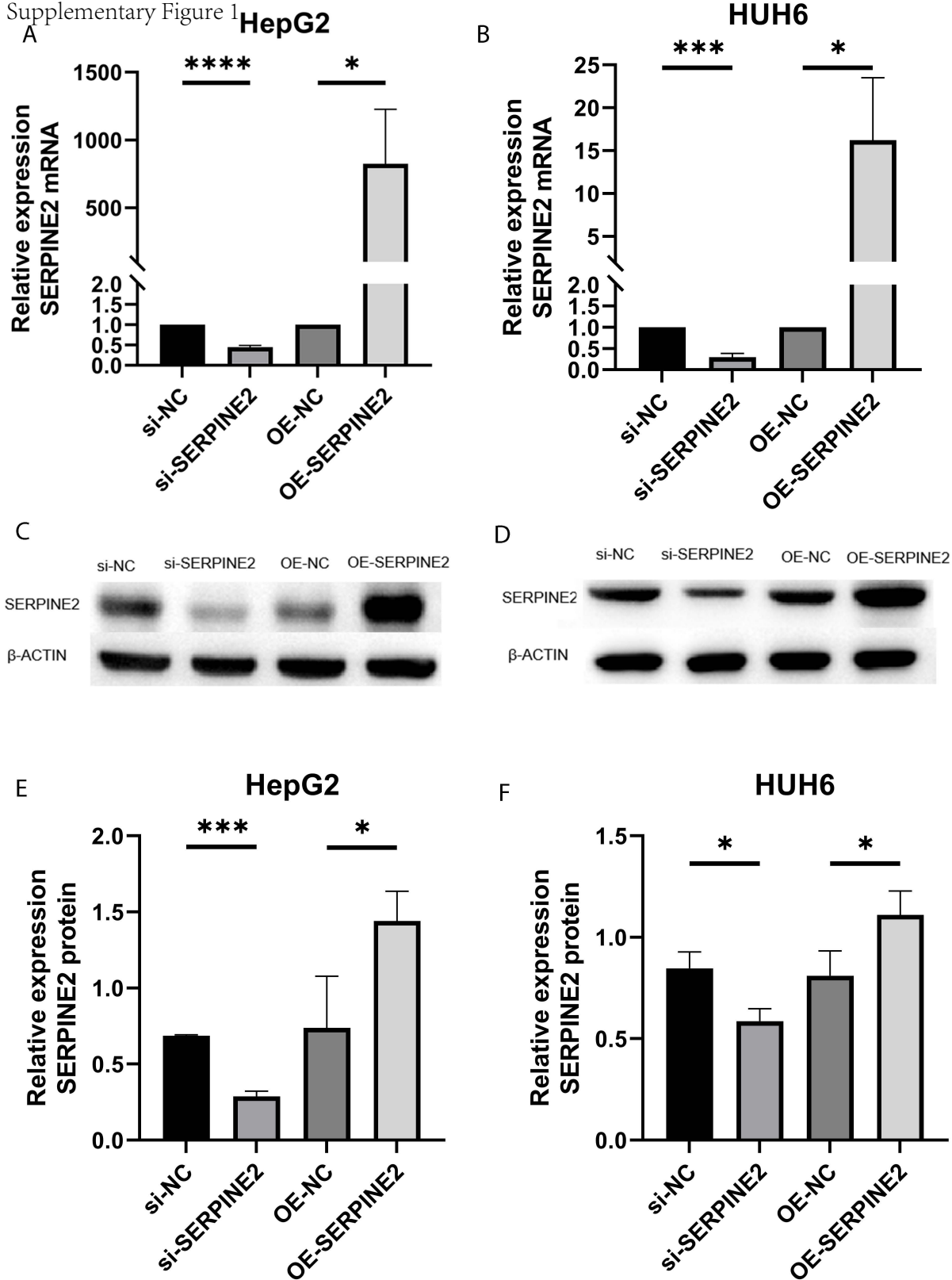

Supplement: Supplementary Materials — Supplementary figure 1: the relative mRNA and protein expression in HepG2 and HUH6 cell lines after being transfected with small interfering RNAs (siRNAs) and overexpressed plasmid against SERPINE2. (A) The relative mRNA expression in HepG2 cell lines after being transfected with small interfering RNAs (siRNAs) and overexpressed plasmid against SERPINE2. (B) The relative mRNA expression in HUH6 cell lines after being transfected with small interfering RNAs (siRNAs) and overexpressed plasmid against SERPINE2. (C, E) The relative protein expression in HepG2 cell lines after being transfected with small interfering RNAs (siRNAs) and overexpressed plasmid against SERPINE2. (D, F) The relative protein expression in HUH6 cell lines after being transfected with small interfering RNAs (siRNAs) and overexpressed plasmid against SERPINE2. [file 2283541.f1.pdf]
